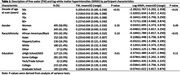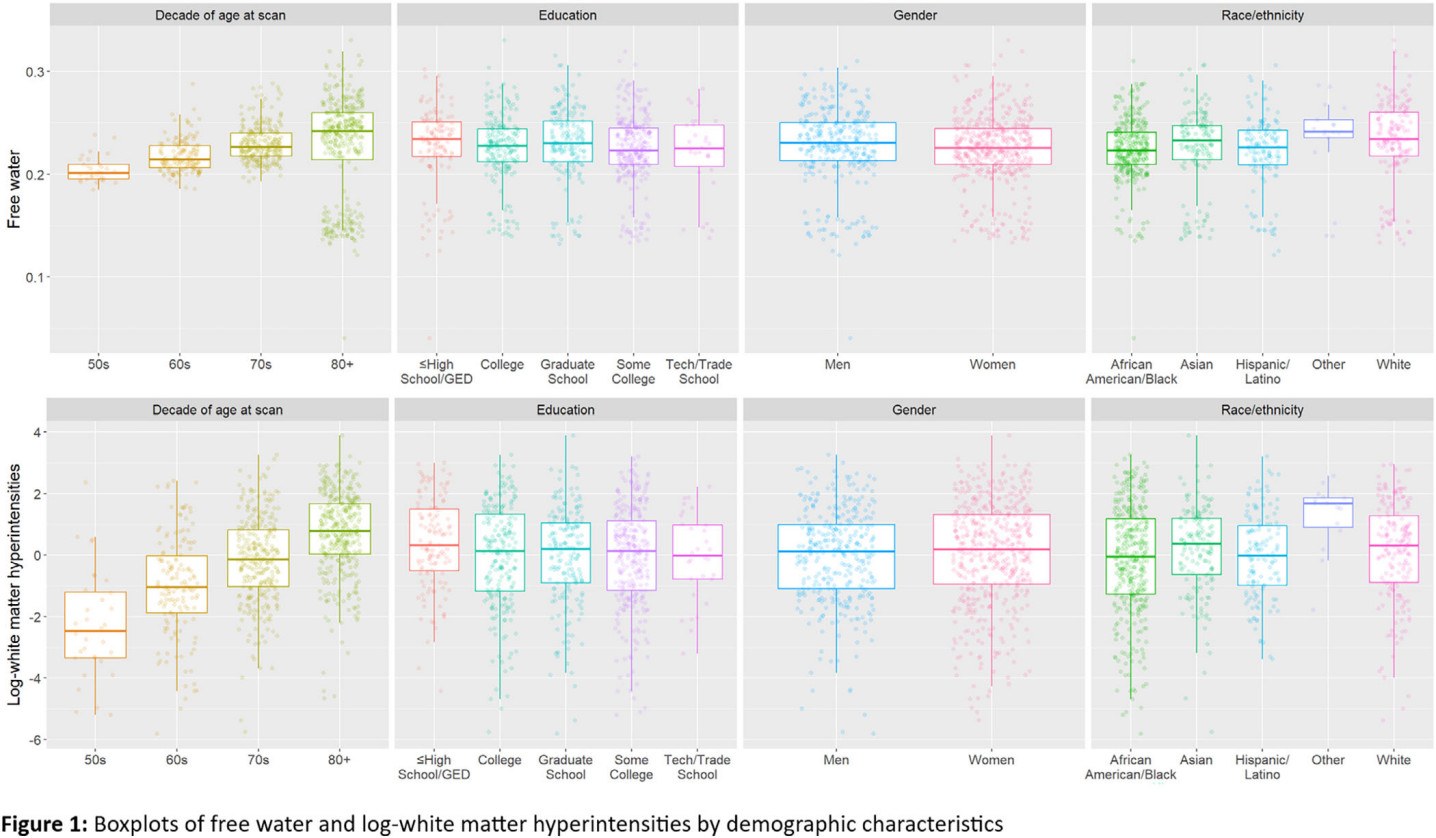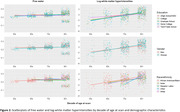# Free water and white matter hyperintensities volumes among diverse individuals aged 54 to 103

**DOI:** 10.1002/alz.095325

**Published:** 2025-01-09

**Authors:** Alexander Ivan B. Posis, Pauline Maillard, Kristen M. George, Paola Gilsanz, Charles S. DeCarli, María M. Corrada, Rachel A. Whitmer

**Affiliations:** ^1^ University of California, Davis, Davis, CA USA; ^2^ Kaiser Permanente Northern California Division of Research, Oakland, CA USA; ^3^ Department of Neurology & Imaging of Dementia and Aging Laboratory, University of California, Davis, Davis, CA USA; ^4^ University of California, Irvine, Irvine, CA USA

## Abstract

**Background:**

Free water (FW) and white matter hyperintensities (WMH) are key biomarkers of white matter integrity yet are understudied in racially/ethnically diverse populations and those over age 80. We describe FW and WMH across age, sex, and education in a diverse cohort.

**Method:**

Data were harmonized from the Kaiser Healthy Aging and Diverse Life Experiences (KHANDLE; mean age = 78±6 years) Study, Study of Healthy Aging in African Americans (STAR; mean age = 69±9 years), and *LifeAfter90* (LA90; mean age = 93±2 years) Study, which are ongoing cohorts of racially/ethnically diverse participants who are long‐term members of Kaiser Permanente Northern California. Participants received neuroimaging via 3T MRI, with harmonized protocols, to measure FW and WMH. WMH was log‐transformed and we calculated residual values accounting for total cranial volume. FW and WMH were described by decade of age at scan, gender, race/ethnicity, and education using mean, standard deviation, and range. Differences in FW and WMH by participant characteristics were tested using analysis of variance tests. We tested the interaction of race/ethnicity with each characteristic in relation to FW or WMH using separate linear regression models.

**Result:**

In 840 participants, median age at imaging was 78 years (range = 54‐103). Fifty‐nine percent were women, 42.3% African American/Black, 18.1% Asian, 16.2% Hispanic/Latino, 21.4% White, and 2% Other. As age increased, FW and WMH increased (p<0.01; Table 1; Figure 1). Participants with ≤High School/GED education had greater FW and WMH. Asian participants had the highest overall mean WMH, followed by White and Hispanic/Latino (p<0.01). However, African American/Black participants generally had greater mean WMH when examined across decade of age at scan (Figure 2). The interaction of gender and race/ethnicity was significant such that African American/Black and Asian women generally had slightly lower mean FW compared to men (p = 0.04). There were no significant interactions between decade of age at scan or education with race/ethnicity.

**Conclusion:**

In this diverse cohort, FW and WMH were associated with increased age, and WMH differed by race/ethnicity and gender. Our findings suggest the utility of FA and WMH among diverse individuals, across a large age range, which is critical for advancing neuroimaging biomarker research.